# Services just for men? Insights from a national study of the well men services pilots

**DOI:** 10.1186/1471-2458-13-425

**Published:** 2013-05-01

**Authors:** Flora CG Douglas, Joe Greener, Edwin van Teijlingen, Anne Ludbrook

**Affiliations:** 1 The Rowett Institute of Health and Nutrition, University of Aberdeen, Aberdeen, UK; 2 Department of Social Work, Care & Justice, Liverpool Hope University, Liverpool, UK; 3 Centre for Midwifery, Maternal & Perinatal Health, University of Bournemouth, Bournemouth, UK; 4 Health Economic Research Unit, University of Aberdeen, Aberdeen, UK

**Keywords:** Men’s health, Health-seeking behaviour, Health promotion, Prevention, Primary care, Qualitative interviews

## Abstract

**Background:**

Men continue to have a lower life expectancy in most countries compared to women. Explanations of this gendered health inequality tend to focus on male risk taking, unhealthy lifestyle choices and resistance to seeking help from health services. In the period 2005–2008 the Scottish Government funded a nationwide community health promotion programme aimed at improving men’s health, called Well Men Service Pilots (henceforth WMS).

**Method:**

This paper explores WMS programme users’ perspectives and experiences of health help-seeking against theories of hegemonic masculinity as explanatory frameworks for men’s behaviour around health and illness, and their views on a male-specific focus of the programme. It is based on a secondary analysis of 43 semi-structured interviews with men who engaged with this programme.

**Results:**

We challenge the commonly held notion of men as being disinterested in their health, and point to their heterogeneity in relation to their views about health and notions of health seeking. Moreover, men in our study were largely ambivalent about the need for gender specific services, despite their positive reactions to the programme in general.

**Conclusions:**

Our findings question the utility of some theories of masculinity that posit somewhat simplistic explanations for men’s reluctance to seek help from formal healthcare services. They also suggest that providing male-specific health services may not significantly address men’s supposed reluctance to seek help from formal health services. Essentially, age seemed to be more important than gender. All encompassing health programmes are likely to fail to meet their health improvement objectives if they attempt to engage with men on the simple basis that they are male.

## Background

Concerns about gendered health inequality have driven a range of recent policy interventions, and the issue of men’s (ill) health has become increasingly discussed in the public sphere [1,2]. Various explanations about the causes of this inequality have emerged, ranging from biological, psychological and socio-political explanations [3]. However, it is the behavioural/cultural explanation that has tended to dominate this debate [4-6]. From this perspective, men’s propensity to die at a younger age than women is commonly explained as a consequence of their deficient behaviour and lifestyle [7]. Men are considered more likely to ‘behave badly’ e.g. engage in excessive alcohol consumption or smoking [8], and fail to seek help (early) from formal health services when symptoms occur [9,10]. In line with this perspective, policy responses in the UK have consequently tended to favour ‘individual-focused’ behaviour change interventions [4,5].

This paper presents a secondary analysis of qualitative data that captured the experiences of men who engaged with a national government programme that was aimed at improving men’s health in Scotland. These data were generated during the external evaluation of this programme that took place between 2006 and 2008 [11]. Our analysis focuses on WMS programme users’ perspectives and experiences of health help-seeking against theories of hegemonic masculinity as explanatory frameworks for men’s (ill) health behaviour, and their views on male-specific focus of the programme. We were unable to explore these themes in depth during the original evaluation due to its time constraints.

### Men’s health inequalities

Across the world, women live approximately six years longer than men [12]. There is also considerable variation in male life expectancy within and between European countries, including the UK. For example, a man born Lithuania in 2011 can expect to live between 68.1 years and 79.9 years if born in Sweden, which represents a range in life expectancy of 11.8 years [13]. Scottish men live on average 2.4 years less than those living in other parts of the United Kingdom (UK); i.e. 75.04 years compared to the national average of 77.53 years in 2008 [14].

### Theories of masculinity and men ‘behaving badly’

Normative understandings of masculinity are often associated with risky behaviours (e.g. drinking, smoking, late health care seeking) even after social class is accounted for [8]. For example, White and Johnson [15] showed that men delayed contacting health services for acute chest pain whilst attempting to rationalise the symptoms. Others have found that men delay or fail to seek help for health problems, even when symptoms occur [9,16]. O’Brien and colleagues found that men resisted visiting doctors for minor problems, and commonly tolerated a degree of pain for some time before visiting their doctor; reflecting (they argued), masculine constructs of ‘hardiness’ [10]. Sanden and colleagues found that men with testicular cancer often delayed visiting health services after finding a lump [17].

Men’s reluctance to seek help is often explained through theories of masculinity [9,10,18]. Masculine health-related behaviour has been conceptualised within social constructionist theory. Men, it is argued, enact dominant masculine behaviours which reflect the socially constructed ‘independent, self-reliant, robust and tough’ male [19]. These social and cultural gendered constructions determine cognitive understandings, behaviours of individuals and health-related practices. Masculinity (and femininity) is articulated within context specific relationships and social environments. In this theory, the resistance to seek help from medical services and engagement in risky behaviour are both associated with a wider understanding of masculinity. Importantly, theories of masculinity rest on the concept of ‘hegemonic’ or dominant masculinity [20]. In debates regarding health-related help-seeking, hegemonic masculinity is thought to create patterns of behaviour which are based on resisting contact with formal services in order to emphasise self-sufficiency and robustness.

However, the notion of a hegemonic masculinity as the dominant force in health-related decision-making has been criticised. Some have suggested that masculinity has become abstracted and consequently divorced from studying the actual practices of men [21]. Masculinity intersects with a number of other statuses including social class, age, and ethnicity [9,18]. In other words, it is impossible to pull men out of the social structures in which they are located, and in many instances other factors, such as social stratification or age, will be more important than gender [1]. One of the theoretical implications of envisaging masculinity as an external pressure is that men are conceptualised as ‘slaves’ to hegemonic discourses. Unfortunately, this perspective has led to ‘men’ being constructed as a universal category [22].

Problems arise when visualising the relationship between masculinity and health-related behaviour using such mono-dimensional perspectives. Men who had experienced major health problems have different attitudes to help-seeking that varied with age [10]. Older men and men who had been through some sort of major health problem were far more likely to be critical of hegemonic masculinities which devalued help-seeking. However, younger men were much more concerned about ‘fitting in’ with their peers and thus more likely to emphasise and reproduce anti-help-seeking forms of masculinity. Ridge and colleagues have also reported that debates about men’s help-seeking in relation to mental health have suffered from this notion of men as a homogenous group [23].

Moreover, there is disconfirming evidence linking gender with health help-seeking in general. The idea that women are more likely to consult their doctor than men is contested [24-26]. MacIntyre et al. contended that men were as likely to consult their general medical practitioner (GP) as women when suffering from five common conditions, with the exception of mental health problems. Smith and colleagues found that men were just as interested in their health as women, but tended to use different criteria to monitor their health [27]. Furthermore, a number of studies have shown that a proportion of women also delay seeking help or contacting health services [28].

### Male-specific health services

Early theories of hegemonic masculinity [29,30] clearly emphasised the situational nature of masculinity, but the subsequent men’s health movements adopted a somewhat simpler version of the theory. For example, some have argued that men’s health-promotion campaigns have suffered from two important fallacies [31]. First, they have largely tended to focus on physical health assessment and lifestyle information to the detriment of dealing with wider issues around gender inequality. Secondly, that they have often employed stereotypical notions of masculinity in their social marketing campaigns, neglecting many men who do not associate with hegemonic ideals of masculinity.

Despite evidence that men and women have distinctly different health concerns, current research does not provide a strong steer on how to design services to address those. The lack of evidence linking male health status with help-seeking fails to clearly indicate what gains in population health can be realised through getting more men to use health services more frequently. Furthermore, male-specific health interventions do not show a clear connection between such services and health outcomes [32,33]. In spite of this, there have been growing demands for more gender specific health services in recent years [34-36].

### Well Men’s services pilots programme

In 2005, the Scottish Government provided funding to support community-based partnerships to develop so-called Well Men’s Services (WMS). Three distinct policy aims underpinned this programme, i.e. to: 1) *‘promote healthier lifestyles and attitudes amongst men’*; 2) *‘provide men with an opportunity to undertake a health assessment and to obtain advice and support on health and lifestyle issues’*; and 3) *‘effectively engage all men’*[11]. The WMS pilots were tasked with identifying effective ways of engaging with all men, and in particular, those so-called ‘hardest to reach’ as a consequence of social exclusion or discrimination.

Sixteen pilot services were located in seven different geographical areas of Scotland ranging from inner-city areas, to rural and remote regions, and were based in primary care centres, pharmacies, workplaces, health information buses and other community locations. Across the pilot areas, there was some variation in what was offered within individual programmes. For example, some areas incorporated a ‘community development’ dimension within their programme. However, almost all pilots offered blood pressure, cholesterol and blood sugar testing, and a body mass index calculation. The pilots were generally delivered by primary care nurses who also provided lifestyle behaviour change advice and/or referral to other statutory or voluntary organisations. In spite of this apparent heterogeneity, there was a common ‘intervention logic’ [37] shared by all but one pilot service. This logic was consistent with the behavioural/cultural explanation of male ill health, i.e. that men’s health would be improved by professional intervention to identify and ‘fix’ their behavioural and physical deficiencies [38].

## Methods

Interviews were conducted with 43 men who had used the WMS programme in three different geographical regions; representing a good spread of participants living in medium to large towns, remote/rural areas and inner city locations. Ethical approval was granted by the Multiple Research Ethics Committee (Scotland A) and informed consent was acquired from all participants.

Interviews were conducted by telephone or in person, and lasted between 20 and 45 minutes. We explored a range of issues regarding their experiences of their WMS pilots, health help-seeking, health beliefs and practices, and male-only health services. The original interviews were semi-structured (based on Grounded Theory approaches) and participants were encouraged to speak freely on topics around the questions [39]. Consequently, data were generated that contained themes relating to male help-seeking more generally, and the notions of masculinity linked to health and well-being which we did not have time to explore in depth during the original study.

Our initial analysis focused on addressing the commissioned evaluation questions which were primarily concerned with finding out what WMS clients liked and did not like about their local pilots. During this secondary analysis we used the raw un-coded transcripts to explore perspectives and experiences of male health help-seeking and opinions about male-specific health services against theories of hegemonic masculinity. There is significant value to be gained from re-analysing data in order to gain more insight and knowledge that can be used to inform contemporary debates [40,41].

## Results

The men who took part in the study ranged in age from 24 years to 79 years. Most interviewees were employed or retired, owned their own home and have some form of post secondary qualification.

The analysis identified three emergent themes, i.e.: (a) the health monitoring opportunity the WMS programme had represented; (b) background anxiety and uncertainty about the seriousness of health concerns; and (c) ambivalence regarding men-only health services. One more exceptional theme was that interviewees endorsed the idea of men-only health services.

### Health monitoring opportunity

We found that interviewees seemed to have had a long-standing interest in their own health, and it appears that the WMS programme had represented an opportunity for some to engage in more self health monitoring. This theme is illustrated in the following two excerpts. The first is from of a much longer participant’s account of the steps he had taken in recent years to address long-standing concerns he had about his weight. This narrative emerged when asked why he had decided to use the WMS programme:

*… because I am quite a healthy guy you know I try to keep myself healthy you know but the weight problems was an issue ‘cause I’ve always carried a bit of weight you know.......but as I say I try and do as much exercise as I possibly can. I bowl three days a week indoors now. The bowling season is starting so I will be into that again. And I cycle …. I did back in the summer and you know the mornings I got up and my cycling and I cycle round about that half a dozen times you know. I keep myself reasonably fit you know. …Regarding my diet. … I loved a good fry up and that you know… every day I had sausages, beans and tattie scones and I was going for all that you know. But now I haven’t touched that last couple of months. I have never had a fry up in last 10 weeks. Oh I had my last fry up it was round about New Year’s day or the day after it or something like that. …That was my last fry up and I have no touched it since and I have changed from high fat butter to margarine.* (res 43, age not specified)

He goes on to talk about the WMS programme fitting in with his intentions to monitor his health in the future, and in his view that the programme should be promoted heavily to encourage other men to take up to help with problems like drug and alcohol addiction.

In the next example, the participant talks about having been involved in a previous community group at a time when he felt unfit. He describes how he had his blood pressure (BP) checked when he became involved with this group, and that this had been the start of his active attempts to stay fit and healthy. He regarded the establishment of his local WMS project as an another opportunity to stay motivated about his health.

*Well I went on an MOT*^a^*programme before it was a dads’ and kids’ night out at a sports centre… We were to play 5 aside sides with lots of teams there and there was an MOT put in place as well. So you could play football then go and see the MOT and that was my first experience. I got my BP and things taken and I felt that I was really unfit at that time. And ever since that night I have basically gave up smoking, went on to patches and I think weaned myself off cigarettes. Talking about a three years ago. And I thought it would a good idea to do the Well Men Clinic a few years later....I’ve been on and off smoking. So I thought best to go back so I can so can maybe help me as well to keep me motivated in men’s health. I go to the gym. I keep myself even more fit now.* (res 3, age 34)

Many participants had experienced a life-threatening health problem, as well as other minor health complaints, in the past. This seems to have left those men sensitised to the possibility of reoccurrence of health problems in the future, and a desire to avoid a relapse or developing another health condition. For instance, respondent 15 (age 68) described how he had two previous bouts of lymphoma (cancer of the immune system). He described how the follow-up checkups after his cancer had made him more ‘aware’ of his health and had since engaged with formal health services more regularly.

Concerns about their weight (illustrated in the 'res 43, age not specified' above) was a commonly cited reason for participants’ engagement with the programme. Many had either been previously informed they were overweight or understood their bodies in this way. Other lifestyle concerns cited as reasons for engaging with the WMS programme included anxieties about tobacco, drugs and alcohol consumption. However, not all participants wished to modify their current lifestyle/behaviour. Paradoxically, many were merely seeking reassurance that they were still healthy, despite these behaviours. For instance, respondent 21 (age 45) had been ‘smoking dope for 30 years’ and wanted to make sure he was still healthy. He believed that quitting smoking marijuana would benefit his health but did not have any specific plans to do so.

In this illustrative excerpt, the participant talks about the fact that the WMS was something that he thought would be useful to himself or someone else (possibly the lady friend her refers to earlier in the interview) to gain reassurance that he had no major health problems:

*What made me? Well I am interested in being a healthy man of course. But I only want good news. So as long as I am feeling alright I was quite happy to go along albeit having to go to (small town in Scotland). I have no real reason to go ..I mean in terms of suffering anything or having. Any complaint or anything like that no nothing like that.. no I had nothing like that as a background no. It was simply that I thought ..well it might be useful to me and/or someone else* (res 43, age not specified)

Interestingly, later in the interview, he talks about his interest in going into things ‘deeper’, suggesting a greater concern about his health than he indicated at the start of the interview:

*And sometime your mind says ah well I would like to know if I got this or I got that I havena got that. It will help me in some things. I mean at the end of the day you could go a lot you know go deeper and test for things and all that you know.*(res 43, age not specified)

Participants also commonly talked about interlinking concerns related to their own ageing or family health problems as reasons for seeking out the WMS programme. Participants also often referred to relatives with a serious illness as being instrumental in motivating them to engage with the programme. Here, a participant talks about using the programme on the basis of a recommendation from his brother, who apparently had had a positive encounter with the programme when he decided to consult WMS programme staff because he knew he had high cholesterol levels:

*It was my brother that mentioned it one night ‘I am going back, my cholesterol was a bit high .... well we’ll cut that back’ and he has right enough. .... Actually he has now actually lost 2½ stone I think and he watches what he is eating. He has cut down on smoking everything. So there is a perfect example* (res 23, age 41)

Also many highlighted ageing as significant motivating factor, illustrated thus:

*I am reaching 40 and I dinna ken* (= do not know)*, I mean I feel fairly fine, fit and healthy. But you dinna* (=don’t) *know what’s going on underneath. It was just a more in the hope of them coming across anything at all like, being diabetic or just any these sort of things, eh. I was kind of looking for that, like a blood test and things like that* (res 2, age 39)

Another concept to emerge within this theme was the interest expressed in ‘testing’ and getting numerical information about their health status. Many indicated that tests for high blood pressure, cholesterol and blood sugar had been a big *‘draw card’* for them. One pilot area did not provide cholesterol testing (but had originally intended to), which had clearly been a great disappointment to men attending this WMS. This next quote from a participant, who professed no specific health concerns during his interview, talked about the salient issues he remembered from his encounter with the WMS programme which centred on information he had received about his blood pressure and cholesterol:

*I went in here and I wanted to maybe just being nosey about myself. ..I mean I found things like cholesterol, BP one or two I can’t remember 2–3 things I found out. That were helpful to me you know and these sort of things . .. it was just sheer nosiness. What went away it was I enjoyed it I mean it wasna all it was was time aye. I just went it was just going there and there was nothing it was just a time factor and I went there aye it was fine. I enjoyed what happened aye.* (res 42, age 24)

The other interesting issue to note in this passage was his enjoyment of the encounter, and his judgement of its usefulness.

General lifestyle advice and signposting men to other services (e.g. social services or voluntary organisations) were major components of all WMS programmes. This information giving was offered alongside the physical testing. However, only a few men talked about gaining advice as strength of WMS, or as a motivation for contacting the programme.

Related to the notion of opportunity, men often cited ease of access to the WMS programme, and low cost (free) as being important factors in encouraging them to take up this opportunity. Views about convenience often centred on the fact that WMS offered services out-of-normal working hours or being in an easily accessibly local area.

Another determinant in men accessing a WMS programme was the encouragement of a partner, spouse or female acquaintance. Wives and girlfriends were often described as ‘nagging’ them to attend. However, there were numerous other examples in our data where it seems that men had been responsible for encouraging other men to use WMS. That is, other men (connected and unconnected with the WMS) had encouraged initial engagement with the programme, with participants themselves encouraging other male friends and relatives to use the programme once they had done so.

#### Background anxiety and uncertainty about the seriousness of a health concern

Related to the dominant theme of health monitoring opportunity, was the theme of uncertainty about the seriousness of existing health concerns. As highlighted above, many men had experienced some form of ill health or life threatening condition that had sensitised them to the idea of avoiding future ill health that was not yet apparent. Seeking comfort about a diverse set of concerns was highly prominent in the data and the phrase *‘peace of mind’* occurred in a quarter of all interviews.

Many also talked about their uncertainty about the status of their health concerns, whether they linked it to their lifestyle, age, specific health problems or family history. Expressions of disquiet that their concerns were not serious enough to take to conventional primary care services were commonplace. Some men expressed anxiety about bringing trivial health matters to a medical practitioner and talked about being keen to avoid wasting people’s time. Some expressed a desire to talk about what they seem to regard as lower level health concerns, to another type of health professional first (usually a nurse) as illustrated here;

*There has just been a couple of wee* (=small) *problems that I have been having. Nothing really major but things I dinna* (=don’t) *feel that I want to waste a doctor’s time wi* (=with)*, but at the same time I would like to get an opinion of somebody who is a nurse or something* (res 24, age 52,)

The next participant talks about what might prevent him seeking advice about his health in the future. He indicates that he wouldn’t feel inhibited about consulting a doctor about a health concern, but suggests that the WMS offered the opportunity to check out lower level health issues with the nurses, but would take serious health concerns to the doctor:

*But you know, there is nothing that I think would stop me coming unless but I think if it was something I felt really worried about healthwise than about mental healthwise but I think I would probably have been going to see my doctor anyway. If it was something that I was concerned about cause you are concerned about your health but no in that manner. This is more just come along I would think come along to see the girls again. ‘oh how you doing etc’ .. Whereas you know, I think if had something seriously or (again I keep saying seriously wrong) but, if I had something I felt I would need a doctor’s advice, I would go to the doctor.* (res 40, age not specified)

Interestingly many participants talked about seeking help from a doctor subsequent to their initial consultation with a WMS nurse. This was commonly linked to the discovery of apparently abnormal blood pressure readings or elevated blood sugar or cholesterol levels. Some men described attending a weight management programme after their WMS consultation, indicating that concerns about their weight were in line with a clinical judgement about the same issue. Others described changes to their lifestyle in relation to diet and exercise routines as a consequence of their checking out their ‘niggling’ concerns. There was also a little discussion about people changing alcohol, drinking or drug taking habits after contacting WMS – but this featured much less frequently than dietary or activity change claims.

#### Ambivalence about male specific health services

The interviews also explored individual motivations for contacting the WMS. Not one single participant mentioned the male-specific nature of the service as an influencing factor in their decision to consult their WMS programme. However, later on in the interviews we probed participants’ views about male-only health services. It is from this direct probing that the following themes emerged.

Most participants expressed indifference about male-specific health services or indeed expressly pointed out that they thought these were not a good idea and did not believe men-only services were worthwhile. This was particularly true for the more rural and remote WMS attendees.

Some men who had engaged with their WMS programme through their workplaces expressed particular concern about women not being allowed access to WMS, illustrated thus:

Well to be honest I think that was a bit unfair because we have a few women in the company that would have liked to do it as well. Although it is mainly men in the construction industry I think it is a little unfair to be honest (res 42, age 24)

Some believed that male-specific programmes might encourage other men to use the service, but were ambivalent about its personal relevance:

*Aye* (=yes)*, I thought it was a good idea, aye…Men they would just rather no go to the GP* (=General Practitioner/family doctor) *and things eh so. So I just thought I would just get the bull by the horns and just go. So I think it is a great idea that it’s particularly for men, aye.*

Interviewer: Do you think it would have made any difference to you if it was for men and women?

Na, I would have used it anyway. (res 25, age 38))

This sentiment cropped up again and again in the interviews, i.e. participants thought there may be value in providing male-specific services for *other* men, but not for them.

A few expressed the view that they thought male health services were a good thing purely because women-only services existed, and that this might help to redress this preceived inequity. A few also thought there could be value in providing a male specific health service to encourage other men use their health services, illustrated by this example:

I can see that possibly the value in that (male-specific health services), I suppose the male sex probably don’t pay as much attention to their health and well being as much as the opposite sex would. Women possibly take a little bit more care of themselves. (Res 33, age 36)

However, most of those who talked about the possible value of such services then admitted that they would have used the WMS even if it had not been targeting men.

A few exceptional cases emerged during this direct probing. One participant claimed that on reflection the male-specific nature of the programme had subconsciously encouraged him to seek it out:

From a psychological point of view I felt it was better than, you know that it was a men’s. You know that it was men’s testing, it felt specific. I didn’t really think that I had contacted it because it was just for men, but thinking about it now I suppose I did. (res 32, age 59)

## Discussion

A range of studies have linked masculine behaviour and beliefs to a reluctance to seek help from formal health services [42]. However, our research joins a growing number of studies which resist simple explanations of men's help seeking (or lack of) as emerging straightforwardly from dominant masculinity [10,22,43]. The notion of a hegemonic masculinity is commonly used as a means of explaining anti-help-seeking attitudes amongst men. Yet studies on help-seeking in general, highlight the reluctance of people to seek medical attention for a whole host of reasons, many of which are difficult to understand in gendered terms [44]. Our findings suggest that the key determinants of help seeking amongst men who had engaged with the WMS related to underlying health concerns and the perceived severity of those health concerns. The influence of significant others, programme marketing and notions of accessibility were also evident but less commonly mentioned.

Indeed, the major theme in this study is that men were not only willing to seek help and advice about current health concerns, but were also generally concerned and sought reassurance about their health when they were not yet experiencing symptoms. They also displayed significant fretfulness about possible consequences of their current lifestyle, or past health scares.

As Smith and colleagues suggested, men appeared to be as concerned about their health as women, but are more interested in their physical/biological health [27]. Participants in this study consistently drew our attention to the clinical health checks as a major attraction of the programme. They wanted 'peace of mind' and reassurance over their own health status and focused on biomedical testing and test results, rather than lifestyle advice or general support as the valued ‘products’ of this programme. The widespread disappointment at not receiving a cholesterol check in one of the larger WMS programmes (as initially promoted) further reflects the men's focus on biomedical aspects of health. These findings concur with Connell’s [29] and White’s [15] conclusions about men’s tendency to view their bodies in a mechanistic manner.

That said, within the context of this study, views about which health concerns to take to a GP varied, and this was a source of anxiety for some men. There was a suggestion that some were concerned not to ‘bother’ the GP with ‘trivial’ health issues and that the GP’s surgery was not the place to seek help about such matters. Some indicated that the WMS had offered them a place to get help about those matters, and cited this as a key strength of the programme. This is consistent with Smith [45] and Galdas [9] who founds that men are less inclined to go to the GP for ‘minor’ health problems. We can only theorise about why our participants felt comfortable approaching a nurse rather than a doctor with such concerns; perhaps fear of losing face or appearing incompetent in front of ‘the doctor’, should those concerns turn out to be unfounded, may provide a partial explanation for this finding. It should be borne in mind that just over 50% of the GP workforce in Scotland is female [46]. Furthermore, other studies have found men to be 'frequent attenders' of GPs [43].

This reticence to make initial contact with a GP about a non-urgent health issue but willingness to engage with another (less threatening?) health professional, merits further exploration. For it is interesting that many WMS attendees often reported having sought help from a doctor subsequent to their WMS consultation. A surprising number of men told stories about how they had discovered or confirmed problems such as high blood pressure, blood sugar or cholesterol levels as a consequence of the WMS visit. As shown earlier, almost all participants reported seeking help from other health services after their WMS consultation.

Indeed, our participants often told stories about help-seeking which did not seem to revolve around their maleness. Perhaps when the men described reasons for engaging with the WMS, such as previous health problems, a family history of heart attacks, or reaching a certain age; that they were engaging in discursive constructions which helped to reinstate their masculinity. However, they did not appear to see their contact with the WMS pilots as related to their status as a man in any sense. They simply were not positioning themselves in relation to an anti-help-seeking masculinity. Furthermore, our findings showed that whilst WMS programme staff had been proactive in seeking out spaces inhabited by men, to engage with them, contacting men directly can encourage them to seek health advice.

While these data suggest that potential gains can be made from encouraging all men to engage with health and other services, they do not necessarily point to the value of male-specific services. Moreover, other such approaches have not had demonstrable impacts on male health outcomes [32]. Very few men referred to the male-specific nature of WMS either as a reason attending WMS, or it having been of any relevance once they had. Moreover, the degree of ambivalence and indifference about the male specific aspect was surprising given the general enthusiasm of our participants attending WMS. Interestingly some men were concerned that women would be disadvantaged by not having access to this programme – once again indicating that their help-seeking was not premised on their maleness. There were a few exceptional cases, suggesting that for a minority of men this may be important. It may be that participants constructed, post hoc, a gender neutral view of their encounter with the WMS programme, but we don’t have data to support that, as we did not explore their prior views of this issue. However, our experience of talking to men face-to-face during the interviews, and informally during the fieldwork suggested that they genuinely lacked concern about the maleness of the service. Indeed, if anything they were more concerned that it should be available as a gender neutral service in order that women would not be disadvantaged. Furthermore, other research has found that some men are ambivalent about the maleness of services, and indeed happy to engage in health promotion activity with female participants [47].

Our findings support the view that conceptualising men as a homogenous group is problematic when designing health improvement interventions [22,48]. Current theory on masculinity and health maintains that men are a heterogeneous group, yet the local WMS programmes were generally aimed at all men, and their success in this aspect was limited [11]. As Smith and Robertson comment ‘[W]when gender has been a central feature of men’s health promotion work it has tended to interpret masculinity simplistically, equating it with a set of (usually negative) characteristics that all men share or a set of common characteristics that all men subscribe to’ (284: 2008).

We argue that continued acceptance of simplistic ‘hegemonic masculinity’ explanations of men’s health behaviours and health outcomes means we continue to reinforce the hegemonic discourse that privileges behavioural explanations of male health inequalities [49,50]. Seeing the world in one rather limited way prevents us from thinking about alternative approaches and solutions within the wider socio-economic and political systems in which men work, rest and play that might bring about health improvement. Providing men-only health services may provide an incentive for some men to consult a health professional earlier in the course of illness, but men’s poor health outcomes cannot be wholly explained by a lack of their interest in their health, or their desire or unwillingness to do something about it.

Finally, it is worth highlighting two potential study limitations. Firstly, the study participants had opted into the WMS programme. Secondly, our respondents were generally older and many had experienced health problems during their lifetime. Older men and men who had experienced some form of illness appear less likely to reproduce hegemonic, anti-help-seeking masculinities [10]. Nevertheless, our study offers important insights for those developing interventions intended to improve men’s health.

## Conclusion

This study indicates that some groups of men are interested and concerned about their current and future health, albeit that their interest was expressed through concerns about bio-physical issues. Their reported behaviour post WMS engagement suggests a direct benefit from interventions which provide men with an opportunity to discuss concerns and anxieties with health professionals that are not doctors. However, as our participants were generally ambivalent about the male-specific character of WMS, it seems that such services do not necessarily need to be provided through formalised male-specific services. Essentially, age seemed to be more important than gender. Also the failure of WMS to reach large numbers of ‘hard-to-reach’ men, suggests that those most in need of preventative intervention may be best tackled through smaller, targeted and group-specific programmes.

Nevertheless we would not want to suggest that public health in the area of men’s health should cease, nor we would want to argue that these endeavours cannot make a serious contribution to improving the lives of men. What is apparent from our findings, however, is that there seems to be little to be gained by jumping from theorisations of hegemonic masculinity as a pervasive norm affecting the health of all men to all-encompassing health programmes which attempt to engage with men on the simple basis that they are male.

## Endnotes

^a^An MOT is a mandatory annual test of car road worthiness that is required by all owners of cars over the age of three years. The name MOT has been adopted as a concept within the men’s health lexicon, and is synonymous with a regular health check up.

## Competing interests

The authors declare that they have no competing interests.

## Authors’ contributions

FD, EvT and AL conceptualised the original evaluation study design. FD and EvT developed the original interview topic guides, and guided the data collection and data analysis for the original study. FD, EvT and JG conducted the majority of the interviews. For this paper, JG led the secondary data analysis and conducted a new review of the literature for it. JG, in conjunction with FD, critically developed the overall argument for the paper and both were responsible for creating early drafts of the paper, with EvT and AL critically commenting on those subsequent drafts till completion of this paper. All authors read and approved the final manuscript.

## Pre-publication history

The pre-publication history for this paper can be accessed here:

http://www.biomedcentral.com/1471-2458/13/425/prepub
